# Development of an integrative database with 499 novel microsatellite markers for *Macaca fascicularis*

**DOI:** 10.1186/1471-2156-10-24

**Published:** 2009-06-05

**Authors:** Atsunori Higashino, Naoki Osada, Yumiko Suto, Makoto Hirata, Yosuke Kameoka, Ichiro Takahashi, Keiji Terao

**Affiliations:** 1 Tsukuba Primate Research Center, National Institute of Biomedical Innovation, 1-1 Hachimandai, Tsukuba, Ibaraki 305-0843, Japan; 2 Department of Biomedical Resources, National Institute of Biomedical Innovation, 7-6-8 Saito-Asagi, Ibaraki, Osaka 567-0085, Japan; 3 Department of Research and Development, Central Blood Institute, Japanese Red Cross Society, 2-1-67 Tatsumi, Koto-ku, Tokyo 135-8521, Japan

## Abstract

**Background:**

Cynomolgus macaques (*Macaca fascicularis*) are a valuable resource for linkage studies of genetic disorders, but their microsatellite markers are not sufficient. In genetic studies, a prerequisite for mapping genes is development of a genome-wide set of microsatellite markers in target organisms. A whole genome sequence and its annotation also facilitate identification of markers for causative mutations. The aim of this study is to establish hundreds of microsatellite markers and to develop an integrative cynomolgus macaque genome database with a variety of datasets including marker and gene information that will be useful for further genetic analyses in this species.

**Results:**

We investigated the level of polymorphisms in cynomolgus monkeys for 671 microsatellite markers that are covered by our established Bacterial Artificial Chromosome (BAC) clones. Four hundred and ninety-nine (74.4%) of the markers were found to be polymorphic using standard PCR analysis. The average number of alleles and average expected heterozygosity at these polymorphic loci in ten cynomolgus macaques were 8.20 and 0.75, respectively.

**Conclusion:**

BAC clones and novel microsatellite markers were assigned to the rhesus genome sequence and linked with our cynomolgus macaque cDNA database (QFbase). Our novel microsatellite marker set and genomic database will be valuable integrative resources in analyzing genetic disorders in cynomolgus macaques.

## Background

Cynomolgus macaques (*Macaca fascicularis*) are one of the most commonly used nonhuman primates in biomedical research. Currently, about two thousand cynomolgus macaques are maintained in Tsukuba Primate Research Center (TPRC), Japan [1]. Several lineages of the captive cynomolgus macaques have genetic disorders such as macular degeneration [2] and endometriosis [3]. In genetic studies, a prerequisite for mapping genes is development of a genome-wide set of microsatellite markers in target organisms. A whole genome sequence and its annotation also facilitate identification of markers for causative mutations. A comprehensive cynomolgus macaque genome database, including a map of Bacterial Artificial Chromosome (BAC) clones, 5'-end expressed sequence tags (ESTs), microsatellite markers, primer sequences for microsatellite markers, and genes around the microsatellite markers would be valuable for linkage analyses, but, unfortunately, complete genome of cynomolgus macaque is not yet sequenced.

A microsatellite marker set is a versatile tool that would assist in colony management, conservation work, and paternity testing of nonhuman primates [4-12]. Microsatellite markers of human [13] and some nonhuman primate species [14-17] are now widely available, facilitating linkage analyses in these species. The first generation of genetic linkage maps of baboons [18,19] and rhesus macaques were developed by Rogers, *et al*. [20]. However, few studies have been conducted on microsatellite markers in cynomolgus macaques [12,21]. In this study, we established 499 microsatellite markers that were covered by pre-identified Bacterial Artificial Chromosome (BAC) clones for cynomolgus macaques. We also developed an integrative cynomolgus macaque genome database with a variety of datasets including marker and gene information that will be useful for further genetic analyses in this species. Advantages of this study are (1) since most of newly developed microsatellite marker loci were covered by the BAC clones, we could search for their chromosomal locations by *in silico *mapping, (2) these microsatellite markers were mapped to the rhesus macaque genome sequence , and (3) the 499 novel markers established in this study outnumber the previously reported microsatellite markers in other macaques and are probably useful for linkage studies in other non-human primate species as well.

At a genome-wide level, the cynomolgus and rhesus macaque genomes are very similar; their genetic divergence is about 0.4% at a nucleotide level [22]. In addition, their karyotypes are also very similar [23]. To design cynomolgus macaque microsatellite markers based on the rhesus macaque genome sequence would be a reasonable and efficient way of establishing a species-specific genomic conformation for this species. The development of a linkage map in this species is a first step toward exploring the genes responsible for genetic disorders in captive macaques.

## Results

### Identification of polymorphic microsatellite and construction of microsatellite marker database for cynomolgus macaque

BAC-end sequences of 768 clones of a cynomolgus macaque were determined. Of these, 487 BAC clones were successfully mapped onto the draft rhesus genome sequence (see method). Within the regions that were covered by the BAC clones, we selected 671 candidate loci from 394 BAC clones that harbor dinucleotide repeats equal or longer than 20 bp in the rhesus genome sequence. Of these, 34 markers were selected from rhesus macaque or human microsatellite markers identified by previous studies [13,24-28]. Our marker set does not contain the markers previously developed by Kikuchi *et al*. [21]. These primer sequences and their genomic locations are presented in Additional file 1.

**Figure 1:**
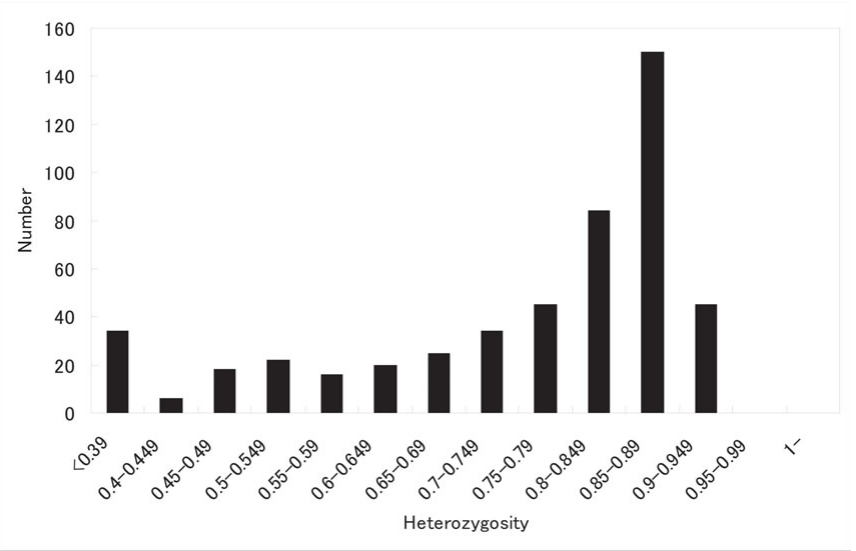
**Distribution of heterozygosity for the 499 microsatellite markers**. Heterozygosities (*H*_*e*_) range from 0.10 to 0.94, with a mean of 0.75. Among 499 polymorphic markers, 324 (64.9%) had *H*_*e *_> 0.75.

Next, we investigated whether these candidate repeats for microsatellite markers are polymorphic using 10 unrelated cynomolgus macaque individuals from Indonesia, Malaysia, and the Philippines. Of the 671 microsatellite markers tested, 499 (74.4%) gave rise to polymorphic PCR products, approximately the same size as expected from the rhesus or human genome sequence. The detailed information is presented in Additional file 1 and also on our website . Because some of the microsatellite markers located on very close loci, which were covered by single BAC clone, we estimated the coverage of the genome by the microsatellite makers using only one polymorphic microsatellite marker which have the distance at least 0.1 Mbp between neighboring markers. The average distance between newly developed markers was about 10 cM, assuming that the macaque genome comprised 3000 Mbp of nucleotides. PCR product sizes are 63–647 bp with an average size of 247 bp. The average number of alleles per polymorphic marker was 8.20 (range 2–17) and the average expected heterozygosity was 0.75 (range 0.10–0.94) for these 499 markers in the cynomolgus macaques. The distribution of expected heterozygosity values showed that a substantial number of the markers have expected heterozygosity greater than 0.80. Microsatellite markers with expected heterozygosity > 0.75 are regarded as highly polymorphic [20]. According to this criterion, 324 of 499 markers (64.9%) were highly polymorphic (Figure 1). In order to check the mode of inheritance of these markers, we investigated additional four families consisting of 27 animals to confirm the inheritance of 453 autosomal markers and found that 412 markers showed no contradictions concerning Mendelian inheritance (see Additional file 2).

We investigated the distribution of the microsatellite markers on the human and rhesus chromosomes. As shown in Table 1, the novel microsatellite markers were distributed over all autosomes and X-chromosome of both species. Since the draft genome sequence of the rhesus Y-chromosome is not available, we did not obtain microsatellite markers on the Y-chromosome.

**Table 1:** Microsatellite marker distribution on rhesus macaque and human chromosomes.

Rhesus chromosome	Human chromosome	Microsatellite number
1	1	37
12	2	17
13	2	30
2	3	35
5	4	27
6	5	28
4	6	25
3	7	36
8	8	18
15	9	10
9	10	22
14	11	22
11	12	33
17	13	27
7	14	12
7	15	15
20	16	15
16	17	10
18	18	18
19	19	8
10	20	7
3	21	7
10	22	14
X	X	26

### Construction of integrative cynomolgus macaque genome database

We further constructed a genome browser for cynomolgus macaques, based on the rhesus genome sequence. In this database, users can search the positions of cynomolgus macaque BAC clones, 5'-end expressed sequence tags (ESTs) and microsatellite markers on the rhesus macaque genome. In addition, human and macaque cDNA sequences and rhesus macaque genes predicted by Ensembl [29] were aligned on the genome. This database is also connected to QFbase, which contains data of more than 130,000 cynomolgus macaque cDNAs [22]. This information will facilitate the search for known or predicted cynomolgus macaque genes near the microsatellite markers and help to narrow down candidate regions for functional genes near these markers identified by linkage analysis. The address of the database is .

## Discussion

At present, a few microsatellite markers have been reported for cynomolgus macaques [12,21] even though the microsatellite tool would be valuable for studying cynomolgus macaque genetic diseases. In this work, we have designed 637 primer pairs and selected 34 primer pairs from NCBI/UniSTS. Of the 671 marker candidates, 499 polymorphic microsatellite markers located on cynomolgus macaque BAC clones. These polymorphic markers could be amplified with high probability with the same protocol as for human microsatellite markers used for other macaques [16,20]. Most of the developed microsatellite markers have high expected heterozygosity and are distributed throughout the human and rhesus macaque chromosomes. Our microsatellite marker set will be available for various studies in macaques, and macaque chromosomal information of developed microsatellite markers is also available from the following database . These BAC clones will be helpful for further identification and functional analysis of genes implicated in genetic disorders.

In the genome database of cynomolgus macaques, we assumed that the synteny of the cynomolgus and rhesus macaques is highly conserved. Although previous studies suggested that their chromosomes were highly similar at a microscopic level, smaller translocations, insertions or deletions may exist between the two genomes [22,23]. The BAC resource can be used to verify such genomic differences by fluorescent *in situ *hybridization (FISH) method. We verified the suitability of such mapping by FISH with 12 BAC clones. Although mapping all BAC clones by FISH is not a practical approach, we are able to check the synteny between the two genomes when a particularly interesting locus is found by further studies (see Additional file 3).

In the polymorphism analysis, all PCR products showed high fluorescence intensities, indicating the presence of ample labeled PCR product, even without optimization. Optimization of annealing temperature and magnesium concentration for the unsuccessful markers using cynomolgus macaque DNA would certainly yield additional useful markers. In addition, many of the microsatellite polymorphisms reported here will also be useful in other macaques [30,31].

Currently, about 800 human microsatellite markers, which cover all areas of the human genome with intervals of 5 cM, are commercially available. Rogers *et al*. developed the first generation of genetic linkage maps of baboons [18,19] and rhesus macaques [20], primarily consisting of human microsatellite loci amplified using the published human PCR primers. Human markers have been tested in the baboons, and over 280 microsatellites were used in studies for osteoporosis in this species [32,33]. The 499 novel markers established in this study outnumber the previously reported microsatellite markers in baboons and rhesus monkeys and are probably useful for linkage studies in other non-human primate species as well. These microsatellite markers are also valuable resources for the management of captive macaque colonies.

We are currently using linkage analysis to identify genetic loci implicated in hereditary macular degeneration in cynomolgus macaques, which is the only animal model of human age-related macular degeneration. Early onset macular degeneration occurred spontaneously in certain cynomolgus macaque families at the Tsukuba Primate Research Center (TPRC), and family analysis revealed that this disease is controlled by autosomal dominant genes [2]. The integration of various genetic tools including our database would greatly facilitate the genetic-based research on disease models in the future. We should note that, however, the whole genome association studies, especially for candidate genes with only minor effects, might require a far denser map than that reported here.

## Conclusion

Cynomolgus macaques (*Macaca fascicularis*) are one of the most commonly used nonhuman primates in biomedical research. In this study, we established 499 microsatellite markers for cynomolgus macaques. We also developed an integrative cynomolgus macaque genome database with a variety of datasets that will be useful for further genetic analyses in this species. The development of a linkage map in this species is a first step toward exploring the genes responsible for genetic disorders in captive macaques. These datasets are definitely valuable to many researchers who are in the field of primate genetics.

## Methods

### DNA sampling and pedigree structure

Whole blood samples were obtained from 37 pedigreed cynomolgus macaques, aged 3–29 years, consisting of 17 males and 20 females housed at the TPRC, National Institute of Biomedical Innovation (NIBIO), Tsukuba, Japan. Blood samples of 10 unrelated individuals (four males and six females) were used for the polymorphism analysis. They consist of three Indonesian, four Philippine, and three Malaysian cynomolgus macaques. Blood samples of 27 individuals in four families (13 males and 14 females) from Malaysian cynomolgus macaques were used for the inheritance analysis. Genomic DNA was isolated from 10 ml of heparinized peripheral blood using the Wizard Genomic DNA purification kit (Promega, WI, USA). These macaques were cared for and handled according to guidelines established by the Institutional Animal Care and Use Committee of the NIBIO and the standard operating procedures for macaques at the TPRC. Collection of the blood was conducted in accordance with all guidelines required in the Laboratory Biosafety Manual, World Health Organization at the TPRC.

### BAC library and BAC-end sequence

We used a BAC library that was constructed using DNA from renal cells of cynomolgus macaques. The library consists of approximately 110,000 recombinant BAC clones providing 3.4-fold coverage of the cynomolgus macaque genome. The cynomolgus macaque BAC library was obtained from the Department of Biomedical Resources, National Institute of Biomedical Innovation, Osaka, Japan. DNA sequencing was performed with BigDye Terminator v3.1 Ready Reaction Mix and ABI Prism-Avant Genetic Analyzer (Applied Biosystems, CA, USA).

### *In silico *mapping of the BAC-end sequences on the rhesus macaquegenome

The BAC-end sequences were mapped onto the draft genome sequence of rhesus macaques (rheMac2 assembly) using a BLAST program (*E *= 10^-30^). Repeat sequences were masked before the BLAST search. When the two BAC-end sequences from the same BAC clone were aligned as head-to-head directions within the range of 10–300 kb on the rhesus genome draft sequences, we assumed that the BAC clone was correctly assigned to the genome. In order to choose candidate loci for microsatellite markers within the region, we surveyed short tandem repeats (STRs) spanning at least 20 nucleotides, with motif length 2 (i.e., CACACA-nucleotide repeats), which was identified using Tandem repeats finder software [34].

### Primer design and PCR

For 637 microsatellite loci on sequenced BAC clones, paired primers were designed using DNASIS software (Hitachi Software Engineering, Tokyo, Japan). The primer sets were confirmed not to match more than one region of the rhesus genome draft sequence [35]. MFA0028-0061 markers were selected from the NCBI/UniSTS id: markers and mapping data are 72106, 147912, 8379, 63879, 13743, 19265, 181717, 37796, 74733, 256705, 75673, 10546, 264996, 153124, 73737, 65585, 148897, 9343, 22659, 42001, 46786, 41570, 78383, 13963, 147924, 83050, 46652, 148707, 11528, 89846, 30829, 94606, 94603, and 76519, respectively [13,24-28]. The forward primers were labeled with one of three fluorescent dyes, 6-FAM, HEX, or NED. Amplification was done in a 384-well format on the 9700 Thermal Cyclers (Applied Biosystems, CA, USA). Ten microliters of the reaction mixture contained 10 ng of genomic DNA, 2.5 nmol each of dATP, dCTP, dGTP, and dTTP, 0.25 units of ExTaq, 5.0 pmol of forward and reverse primers, and the manufacturer's PCR buffer (all purchased from Takara Biosystems, Otsu, Japan). Cycling conditions are as follows: an initial denaturation was performed at 94°C for 5 min, 30 cycles of amplification were performed at 94°C for 1 min, 55°C for 1 min, and 72°C for 1 min, and one cycle of extension was performed at 72°C for 7 min. The same PCR conditions were applied to all amplifications.

### Analyses of microsatellite polymorphisms and genetic inheritances

Amplified PCR products were mixed with gel-loading cocktails containing deionized formamide and labeled size standards (GeneMapper-LIZ 500; Applied Biosystems, CA, USA). Samples were run on the Applied Biosystems 3730 DNA analyzer (Applied Biosystems, CA, USA). Expected heterozygosity (*H*_*e*_) of loci was calculated using the formula: *H*_*e *_= 1 - ∑*p*^2^_*i *_[36]. We examined expected heterozygosity according to Nei [37]. Using 453 autosomal markers, genetic inheritance analyses were performed by checkfam [38].

### Database construction

The cynomolgus macaque genome database was constructed using the rhesus genome sequence (rheMac2 assembly) as a reference. Information on microsatellite markers, BAC clones, 130,000 5'-end expressed sequence tags (ESTs) and annotation of genes was visualized with Generic Genome Browser (GBrowse) software [39]. The annotation of human and macaque transcripts on the genome was retrieved from the UCSC genome browser [40].

## Availability and requirements

Database name: QFbase-GBrowse

Database home page: 

Any restrictions to use by non-academics: no restriction

## Authors' contributions

AH, NO, YS, and KT contributed to the designing of the research. AH, NO, YS, YK, and IT performed the experiments and analyzed the data. NO and MH contributed to the database construction. AH, NO, YS, and KT wrote the manuscript. All authors read and approved the final manuscript.

## Supplementary Material

Additional file 1 **List of 671 primer pairs for microsatellite candidate loci**. Polymorphism was determined with ten unrelated macaques. Chr, Chromosome; Y, amplification; N, no amplification; P, polymorphic; M, monomorphic. These data are also available at our website , and these microsatellite markers are mapped to the rhesus macaque genome database . MFA0028-0061 markers are reported as NCBI/UniSTS id: markers and mapping data are 72106, 147912, 8379, 63879, 13743, 19265, 181717, 37796, 74733, 256705, 75673, 10546, 264996, 153124, 73737, 65585, 148897, 9343, 22659, 42001, 46786, 41570, 78383, 13963, 147924, 83050, 46652, 148707, 11528, 89846, 30829, 94606, 94603, and 76519, respectively. Click here for file

Additional file 2 **Allele data of 453 polymorphic markers**. We checked the mode of inheritance of 453 microsatellite markers in four families (13 males and 14 females) from Malaysian cynomolgus macaques. Of these, 412 markers showed no contradictions concerning Mendelian inheritance. Click here for file

Additional file 3 **Chromosomal localization of cynomolgus macaque BAC clones**. Chromosome numbers are (A): 11, 12, (B): 17, 13, (C): 7, 15, and (D): 19, 19, macaques (cynomolgus and rhesus) and human, respectively. Green spots indicate hybridization signals of BAC DNA. Background staining (red) is propidium iodide (PI). Click here for file

## References

[B1] Suzuki MT, Ono T, Kohno M, Ogawa H (1990). Hour of Delivery in Cynomolgus Monkeys Under Indoor Individually-caged Conditions. Primates.

[B2] Suzuki MT, Terao K, Yoshikawa Y (2003). Familial early onset macular degeneration in cynomolgus monkeys (*Macaca fascicularis*). Primates.

[B3] Ami Y, Suzaki Y, Goto N (1993). Endometriosis in cynomolgus monkeys retired from breeding. J Vet Med Sci.

[B4] Smith DG (1982). Use of genetic markers in the colony management of nonhuman primates: a review. Lab Anim Sci.

[B5] Inoue M, Takenaka O (1993). Japanese Macaque Microsatellite PCR Primers for Paternity Testing. Primates.

[B6] Kanthaswamy S, Smith DG (1998). Use of Microsatellite Polymorphisms for Paternity Exclusion in Rhesus Macaques (*Macaca mulatta*). Primates.

[B7] Nurnberg P, Sauermann U, Kayser M, Lanfer C, Manz E, Widdig A, Berard J, Bercovitch FB, Kessler M, Schmidtke J, Krawczak M (1998). Paternity assessment in rhesus macaques (*Macaca mulatta*): multilocus DNA fingerprinting and PCR marker typing. Am J Primatol.

[B8] Smith DG, Kanthaswamy S, Viray J, Cody L (2000). Additional highly polymorphic microsatellite (STR) loci for estimating kinship in rhesus macaques (*Macaca mulatta*). Am J Primatol.

[B9] Oka T, Takenaka O (2001). Wild Gibbon's Parentage Tested by Non-invasive DNA Sampling and PCR-amplified Polymorphic Microsatellites. Primates.

[B10] Lavergne A, Catzeflis F, Lacote S, Barnaud A, Bordier M, Mercereau-Puijalon O, Contamin H (2003). Genetic analysis of the *Saimiri *breeding colony of the Pasteur Institute (French Guiana): development of a molecular typing method using a combination of nuclear and mitochondrial DNA markers. J Med Primatol.

[B11] Andrade MCR, Penedo MCT, Ward T, Silva VF, Bertolini LR, Roberts JA, Leite JPG, Cabello PH (2004). Determination of genetic status in a closed colony of rhesus monkeys (*Macaca mulatta*). Primates.

[B12] Kanthaswamy S, Satkoski J, George D, Kou A, Erickson BJ-A, Smith DG (2008). Interspecies Hybridization and the Stratification of Nuclear Genetic Variation of Rhesus (*Macaca Mulatta*) and Long-Tailed Macaques (*Macaca Fascicularis*). Int J Primatol.

[B13] Dib C, Faure S, Fizames C, Samson D, Drouot N, Vignal A, Millasseau P, Marc S, Hazan J, Seboun E, Lathrop M, Gyapay G, Morissette J, Weissenbach J (1996). A comprehensive genetic map of the human genome based on 5,264 microsatellites. Nature.

[B14] Chu J-H, Wu H-Y, Yang Y-J, Takenaka O, Lin Y-S (1999). Polymorphic Microsatellite Loci and Low-invasive DNA Sampling in *Macaca cyclopis*. Primates.

[B15] Nair S, Ha J, Rogers J (2000). Nineteen New Microsatellite DNA Polymorphisms in Pigtailed Macaques (*Macaca nemestrina*). Primates.

[B16] Hadfield RM, Pullen JG, Davies KF, Wolfensohn SE, Kemnitz JW, Weeks DE, Bennett ST, Kennedy SH (2001). Toward developing a genome-wide microsatellite marker set for linkage analysis in the rhesus macaque (*Macaca mulatta*): identification of 76 polymorphic markers. Am J Primatol.

[B17] Rogers J, Bergstrom M, Garcia R, Kaplan J, Arya A, Novakowski L, Johnson Z, Vinson A, Shelledy W (2005). A panel of 20 highly variable microsatellite polymorphisms in rhesus macaques (*Macaca mulatta*) selected for pedigree or population genetic analysis. Am J Primatol.

[B18] Rogers J, Witte SM, Kammerer CM, Hixson JE, MacCluer JW (1995). Linkage mapping in *Papio *baboons: conservation of a syntenic group of six markers on human chromosome 1. Genomics.

[B19] Rogers J, Mahaney MC, Witte SM, Nair S, Newman D, Wedel S, Rodriguez LA, Rice KS, Slifer SH, Perelygin A, Slifer M, Palladino-Negro P, Newman T, Chambers K, Joslyn G, Parry P, Morin PA (2000). A genetic linkage map of the baboon (*Papio hamadryas*) genome based on human microsatellite polymorphisms. Genomics.

[B20] Rogers J, Garcia R, Shelledy W, Kaplan J, Arya A, Johnson Z, Bergstrom M, Novakowski L, Nair P, Vinson A, Newman D, Heckman G, Cameron J (2006). An initial genetic linkage map of the rhesus macaque (*Macaca mulatta*) genome using human microsatellite loci. Genomics.

[B21] Kikuchi T, Hara M, Terao K (2007). Development of a microsatellite marker set applicable to genome-wide screening of cynomolgus monkeys (*Macaca fascicularis*). Primates.

[B22] Osada N, Hashimoto K, Kameoka Y, Hirata M, Tanuma R, Uno Y, Inoue I, Hida M, Suzuki Y, Sugano S, Terao K, Kusuda J, Takahashi I (2008). Large-scale analysis of *Macaca fascicularis *transcripts and inference of genetic divergence between *M. fascicularis *and *M. mulatta*. BMC Genomics.

[B23] Dutrillaux B, Biemont MC, Viegas-Pequignot E, Laurent C (1979). Comparison of the karyotypes of four Cercopithecoidae: *Papio papio, P. anubis, Macaca mulatta*, and *M. fascicularis*. Cytogenet Cell Genet.

[B24] Weber JL, May PE (1989). Abundant class of human DNA polymorphisms which can be typed using the polymerase chain reaction. Am J Hum Genet.

[B25] Marineau C, Rouleau GA (1992). Dinucleotide repeat polymorphism at the human CRYB2 gene locus (22q11.2). Nucleic Acids Res.

[B26] Weissenbach J, Gyapay G, Dib C, Vignal A, Morissette J, Millasseau P, Vaysseix G, Lathrop M (1992). A second-generation linkage map of the human genome. Nature.

[B27] Gyapay G, Morissette J, Vignal A, Dib C, Fizames C, Millasseau P, Marc S, Bernardi G, Lathrop M, Weissenbach J (1994). The 1993–94 Genethon human genetic linkage map. Nat Genet.

[B28] Feinstein E, Druck T, Kastury K, Berissi H, Goodart SA, Overhauser J, Kimchi A, Huebner K (1995). Assignment of DAP1 and DAPK – genes that positively mediate programmed cell death triggered by IFN-gamma – to chromosome regions 5p12.2 and 9q34.1, respectively. Genomics.

[B29] Hubbard T, Barker D, Birney E, Cameron G, Chen Y, Clark L, Cox T, Cuff J, Curwen V, Down T, Durbin R, Eyras E, Gilbert J, Hammond M, Huminiecki L, Kasprzyk A, Lehvaslaiho H, Lijnzaad P, Melsopp C, Mongin E, Pettett R, Pocock M, Potter S, Rust A, Schmidt E, Searle S, Slater G, Smith J, Spooner W, Stabenau A, Stalker J, Stupka E, Ureta-Vidal A, Vastrik I, Clamp M (2002). The Ensembl genome database project. Nucleic Acids Res.

[B30] Blanquer-Maumont A, Crouau-Roy B (1995). Polymorphism, monomorphism, and sequences in conserved microsatellites in primate species. J Mol Evol.

[B31] Domingo-Roura X, Lopez-Giraldez T, Shinohara M, Takenaka O (1997). Hypervariable microsatellite loci in the Japanese macaque (*Macaca fuscata*) conserved in related species. Am J Primatol.

[B32] Rogers J, Hixson JE (1997). Baboons as an animal model for genetic studies of common human disease. Am J Hum Genet.

[B33] Morin PA, Mahboubi P, Wedel S, Rogers J (1998). Rapid screening and comparison of human microsatellite markers in baboons: allele size is conserved, but allele number is not. Genomics.

[B34] Benson G (1999). Tandem repeats finder: a program to analyze DNA sequences. Nucleic Acids Res.

[B35] The UCSC Genome Browser Database. http://genome.ucsc.edu/.

[B36] Nei M, Roychoudhury AK (1974). Sampling variances of heterozygosity and genetic distance. Genetics.

[B37] Nei M (1987). Molecular evolutionary genetics.

[B38] Saito M, Saito A, Kamatani N (2002). Web-based detection of genotype errors in pedigree data. J Hum Genet.

[B39] Stein LD, Mungall C, Shu SQ, Caudy M, Mangone M, Day A, Nickerson E, Stajich JE, Harris TW, Arva A, Lewis S (2002). The generic genome browser: a building block for a model organism system database. Genome Res.

[B40] Karolchik D, Baertsch R, Diekhans M, Furey TS, Hinrichs A, Lu YT, Roskin KM, Schwartz M, Sugnet CW, Thomas DJ, Weber RJ, Haussler D, Kent WJ (2003). The UCSC Genome Browser Database. Nucleic Acids Res.

