# Supplementary material for: Development of an integrative database with 499 novel microsatellite markers for Macaca fascicularis
Source: BMC Genet. 2009 Jun 5;10:24. doi: 10.1186/1471-2156-10-24 (PMC2702342; doi:10.1186/1471-2156-10-24)
Supplement: Additional file 3 — Chromosomal localization of cynomolgus macaque BAC clones. Chromosome numbers are (A): 11, 12, (B): 17, 13, (C): 7, 15, and (D): 19, 19, macaques (cynomolgus and rhesus) and human, respectively. Green spots indicate hybridization signals of BAC DNA. Background staining (red) is propidium iodide (PI). [file 1471-2156-10-24-S3.doc]

## Chromosomal localization of cynomolgus macaque BAC DNA on their chromosomes

To test our *in silico* mapping accuracy, we selected twelve BAC clones mapped on different rhesus macaque chromosomes by BAC-end *in silico* mapping and subjected them to fluorescence *in situ* hybridization (FISH) analysis with metaphase cells from peripheral blood lymphocytes of a male cynomolgus macaque according to a standard procedure for the direct R-banding FISH technique (Suto Y, Ishikawa Y, Hyodo H, Uchikawa M, Juji T: **Gene organization and rearrangements at the human Rhesus blood group locus revealed by fiber-FISH analysis.** *Hum Genet* 2000, **106:**164-171).

Each probe DNA was labeled with biotin (Invitrogen), and hybridization signals were amplified using the combinations of rabbit-antibiotin and FITC-goat-antirabbit IgG (Enzo). Human Cot-1 DNA (Invitrogen) was used as a competitor. Slides were counterstained with PI (Vysis), and the hybridization signals were detected with a fluorescence microscope (BX61, Olympus) equipped with a UPlan Apo 100 × (N. A. 0.5–1.35) oil objective and appropriate filter sets. More than five signal images per clone were analyzed with a Power Macintosh G4 (Apple Computer Inc.) equipped with a CCD camera (SPOT, Diagnostic Instruments Inc.) and IPLab v. 3.5.1 (Scanalytics Inc.).

In order to confirm the synteny between cynomolgus and rhesus macaque chromosomes, we selected 12 BAC clones and mapped them onto cynomolgus chromosomes by fluorescence *in situ* hybridization (FISH). All 12 clones were found to hybridize to corresponding chromosomes of rhesus macaques. These 12 clones covered half of the chromosomes (number; 2, 3, 5, 6, 7, 8, 11, 13, 14, 15, 17, and 19) of rhesus macaques.


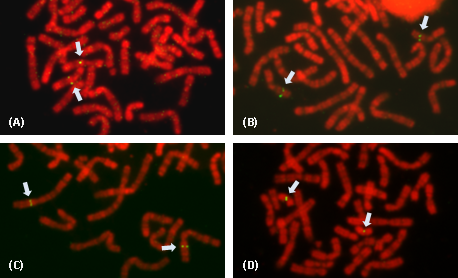


Chromosome numbers are (A): 11, 12, (B): 17, 13, (C): 7, 15, and (D): 19, 19, macaques (cynomolgus and rhesus) and human, respectively. Green spots indicate hybridization signals of BAC DNA. Background staining (red) is propidium iodide (PI).
